# A study on transition shock among first-year residents in China: survey data based on latent profile analysis

**DOI:** 10.3389/fmed.2025.1716120

**Published:** 2025-12-11

**Authors:** Di Xue, Huaijie Yang, Jian Zheng, Aijun Zhu

**Affiliations:** 1 The First College of Clinical Medical Science, China Three Gorges University, Yichang, China; 2 College of Medicine and Health Sciences, China Three Gorges University, Yichang, China; 3 Yichang Central People’s Hospital, Yichang, China

**Keywords:** residents, standardized residency training, transition shock, latent profile analysis, professional identity

## Abstract

**Objective:**

This study aims to identify heterogeneous subgroups of first-year residents experiencing transition shock using latent profile analysis (LPA) and to explore the predictive effects of various dimensions of professional identity on different transition shock types.

**Methods:**

A multi-center, cross-sectional design was employed. From September 2023 to August 2024, a total of 766 first-year residents were selected via cluster sampling from four national-level training bases in Hubei Province, China, for a cross-sectional survey. The survey was conducted using the revised Transition Shock Scale (Cronbach’s *α* = 0.862) and the Professional Identity Scale (Cronbach’s *α* = 0.879). Data analysis was performed using R software, involving latent profile analysis and multinomial logistic regression.

**Results:**

A total of 574 valid questionnaires were returned. Latent profile analysis identified three latent classes: a low psychological-sociocultural shock group (13.41%, *n* = 77), a high physical-knowledge/skill shock group (27.01%, *n* = 155), and a moderate transition shock group (59.58%, *n* = 342). When compared to the low psychological-sociocultural shock group, the various dimensions of professional identity exhibited a contradictory predictive effect for the high physical-knowledge/skill shock group. Specifically, higher scores in the dimensions of professional cognition (OR = 0.724, 95% CI = 0.639–0.820), professional commitment (OR = 0.791, 95% CI = 0.636–0.984), and professional expectation (OR = 0.725, 95% CI = 0.586–0.898) were associated with a greater likelihood of belonging to the low psychological-sociocultural shock group. Conversely, a higher score in professional values was associated with an increased risk of belonging to the high physical-knowledge/skill shock group (OR = 1.139, 95% CI = 1.014–1.279). Using the low psychological-sociocultural shock group as the reference, residents who were Master of Medicine (MM) degree candidates were significantly more likely to be classified into the high physical-knowledge/skill shock group (OR = 3.477, 95% CI = 1.707–7.086). When using the moderate transition shock group as the reference, the same MM degree candidates were less likely to belong to the low psychological-sociocultural shock group (OR = 0.447, 95% CI = 0.244–0.818).

**Conclusion:**

Transition shock among first-year residents exhibits significant heterogeneity. These findings provide evidence for developing targeted intervention strategies. Higher levels of professional cognition, commitment, and expectation are associated with lower levels of transition shock. However, a strong sense of professional values is associated with higher transition shock, a relationship potentially mediated by an idealism-reality gap. It is recommended that tiered competency-building interventions should be implemented for the high physical-knowledge/skill shock group, and a dual-track support system should be designed for Master of Medicine degree candidates.

## 1 Introduction

Global medical education systems are continuously evolving, with countries adopting diverse strategic approaches to meet changing societal developments and healthcare demands. In China, the physician training system follows a three-stage continuum: undergraduate medical education, postgraduate education, and continuing professional development (1, 2). The standardized residency training system serves as the core component of postgraduate medical education in China. Following the completion of a five-year undergraduate medical education (the basic degree requirement), medical graduates undertake a three-year systematic and standardized training program at designated training bases as resident physicians, primarily focused on enhancing clinical competencies (3). The training bases are exclusively comprised of eligible Class A tertiary hospitals—either comprehensive or specialized—that have been officially accredited by the National Health Commission (4). This system was formally initiated in 2013 through a national policy mandate (2). Since 2015, China has integrated professional Master of Medicine (MM) degree programs with standardized residency training, adopting a unified training model that implements a “four-certificates-in-one” system (encompassing the master’s graduation certificate, master’s degree certificate, standardized training certificate, and medical practitioner license). Trainees in the program are primarily categorized into four distinct types, which exhibit certain differences in their educational backgrounds, learning objectives, and organizational affiliations (5–8): (a) Non-affiliated resident trainees: These are graduates who have completed undergraduate medical education or higher but have not signed employment contracts with any medical institution. They are required to seek employment independently after completing the training. (b) In-house resident trainees are official employees of the training base hospital who have not previously undergone standardized residency training or require retraining. They remain fully affiliated with the training base hospital throughout the program and, upon completion, return to their original positions or transition to work in the specialty in which they received training. (c) Externally commissioned resident trainees: These are practicing physicians from non-training-base medical institutions who have not undergone standardized residency training or require retraining. They are commissioned to the training base for standardized residency training and remain affiliated with their original institutions. Upon completion, they must return to their commissioning units. (d) Professional Master of Medicine degree candidates: Their student records are managed by their respective medical universities, while the three-year standardized training is administered by the training base. The residency training and postgraduate education are conducted concurrently. Upon completion of the standardized residency training, they are required to seek employment independently. These trainees hold a dual capacity as both students and resident physicians, necessitating the simultaneous fulfillment of academic requirements and clinical training obligations. Despite the diversity in trainee backgrounds, all residents undergo a standardized three-year training program at the training bases with unified curricula, departmental rotations, and procedural assessments. All residents who have obtained their medical practitioner license, upon completing at least 33 months of rotations and passing the national final examination, receive the essential “standardized residency training certificate.” This certificate is a prerequisite for applying for intermediate technical professional qualifications in clinical medicine specialties (9).

The transition from medical student to practicing physician represents a critical challenge in medical education, a process often accompanied by significant psychological distress and professional identity confusion. A global meta-analysis indicates that the overall burnout rate among residents reaches 51%, with rates in certain specialties exceeding 70% (10). Data from China revealed a 28.3% prevalence of depressive symptoms among residents (11), with a burnout rate as high as 71.4% (12). Additionally, 47.87% of residents reported high turnover intention and a strong desire to leave the profession, indicating severe mental health risks and career challenges faced by this group (13, 14). The mental health issues and high attrition rates within the resident population urgently require attention and should be addressed through evidence-based, strategic support systems.

Professional identity, rooted in Erikson’s theory of ego identity, refers to an individual’s self-concept within quasi-professional contexts. It represents a gradually developed and clarified state of one’s professional role through personal experiences (15), serving as a crucial psychological foundation in professional settings and a motivational force in high-pressure environments (16, 17). Strengthening professional identity is recognized as one of the key strategies to reduce attrition among medical students, as it is closely linked to promoting high-quality training completion, lowering turnover intention, and fostering the development of competent physicians (16, 18). Our previous study found that the professional identity of newly enrolled residents is influenced by transition shock (19). Transition shock refers to the phenomenon of cognitive dissonance and emotional maladjustment triggered by the interactive effects of multiple factors—such as the reconstruction of role positioning, updating of knowledge systems, expansion of responsibility scopes, and reorganization of interpersonal relationships—when an individual transitions from an established role to a new professional role (20). It often manifests as multidimensional symptoms including professional disorientation, shaken professional efficacy, and identity crisis (21–24), exerting significant impacts on psychological well-being and career development (23, 25, 26). This concept closely aligns with the job demands-resources (JD-R) model in professional psychology. The model posits that when job demands (negative factors that consume an individual’s energy, such as work overload, role conflict, and job insecurity) exceed an individual’s resources (positive factors in the work environment, such as support, autonomy, and performance feedback), it can easily lead to physical and mental health depletion and job burnout. Conversely, job resources can buffer the detrimental effects of high job demands (27).

The standardized residency training is fundamentally designed to facilitate the critical role transition of medical graduates from “medical students” to “practicing physicians” (28, 29). This transition process involves not only the systematic development of clinical competency but also the internalization of medical professional values and the reshaping of professional identity. Standardized residency training possesses a transitional nature (30) and is also recognized as a crucial component of professional socialization (31). This represents a critical period highly susceptible to demands-resources imbalance. While previous research has focused on the transition challenges faced by medical students becoming physicians (32) and the transition shock experienced by intern nurses (33), no study has specifically examined the transition shock levels during the standardized residency training phase—a critical developmental transition in the physician growth pathway. Building upon this foundation, the present study specifically focuses on first-year residents, aiming to move beyond a unidimensional quantitative assessment of transition shock by introducing latent profile analysis (LPA). The primary objective is to investigate whether heterogeneous characteristics exist in this population’s response patterns to transitional stressors. As an emerging person-centered statistical approach, LPA can overcome the limitations of traditional variable-centered methods by identifying subgroups with similar response patterns through latent class modeling (34). Compared to traditional cluster analysis, LPA utilizes probabilistic classification and determines the optimal classification solution based on model fit indices, demonstrating unique advantages in researching heterogeneity within the domain of professional psychology (35–37).

This study innovatively introduces the LPA method into research on resident professional adaptation. By establishing a latent class model of transition shock, it aims to understand the combined characteristics of multidimensional symptoms of transition shock among newly enrolled residents and to analyze influencing factors across different transition shock types. This will provide an evidence-based foundation for implementing precise psychological support programs. The anticipated outcomes are expected to deepen the theoretical understanding of the physician professional socialization process and, at a practical level, offer a scientific framework for constructing a tiered and classified support system within standardized residency training.

## 2 Objectives and methods

### 2.1 Study population

This study employed a multi-center, cross-sectional design. First-year residents were recruited via cluster sampling from four national-level standardized residency training bases in Hubei Province, China. Inclusion criteria were: ① registered resident physicians currently in training; ② training duration ≤12 months. Exclusion criteria were: ① non-practicing resident physicians; ② second-year or third-year residents, intern physicians, and other healthcare personnel within the hospitals; ③ those with a confirmed previous history of mental disorders or currently receiving psychological treatment, as verified through admission health screenings, interviews, and self-reporting. Informed consent was obtained from all participants. The sample size was calculated based on the principle that regression analysis requires a sample size at least 5–10 times the number of independent variables. Accounting for a 10% sampling error and to ensure model accuracy and test power, a minimum of 200 cases was required. This study was approved by the Hospital Ethics Committee (Ethics Approval No. 2024–283-01).

### 2.2 Survey instruments

#### 2.2.1 General information of residents

Collected data included gender, age, ethnicity, training status, and marital status.

#### 2.2.2 Transition Shock Scale

This study employed the Transition Shock Scale developed by Xue et al. (33, 38). The scale comprises 27 items across four dimensions: physical (6 items, referring to the state of near-total energy depletion resulting from striving to meet excessively high role expectations from others), psychological (8 items, referring to adverse reactions manifested during the transition period, such as high-intensity tension, emotional instability, and persistent anxiety), knowledge and skills (5 items, referring to the inability to perform normal job duties during the transition due to insufficient personal reserves of professional knowledge and skills), and sociocultural and developmental (8 items, referring to the confusion and unclear positioning experienced during the transition period influenced by factors such as the current social environment, work culture, and work atmosphere). A 5-point Likert scale was used for scoring (ranging from 1 = strongly disagree to 5 = strongly agree), with higher scores indicating greater levels of transition shock. Based on the original scale, cultural adaptation and item optimization were first conducted. An expert panel comprising administrators, educators, and clinical supervisors from standardized residency training programs was invited to evaluate the items for semantic clarity, cultural relevance, and applicability within the resident population, thereby ensuring the scale’s good content validity. In the research team’s preliminary study, this adapted scale had been applied to a resident cohort. Following exploratory factor analysis, the number of dimensions in the scale remained unchanged, but the dimensional assignment of specific items was adjusted: Item 13, originally belonging to the psychological dimension, was reclassified under the knowledge and skills dimension. Confirmatory factor analysis demonstrated favorable psychometric properties for the revised scale. In this study, the scale’s Cronbach’s *α* coefficient was 0.862.

#### 2.2.3 Professional Identity Scale

The Professional Identity Scale for medical students, compiled by Zhang (16, 32), was used to investigate individuals’ overall perceptions toward the goals, social value, and other factors of their intended or current profession. The scale comprises 38 items across six dimensions: professional cognition (7 items, referring to medical students’ understanding and awareness of the nature and significance of the medical profession), professional emotion (7 items, referring to medical students’ emotional investment in the medical profession and whether the emotional experiences derived from the profession are positive or negative), professional commitment (3 items, referring to medical students’ investment in the profession and internalization of social norms leading to reluctance to change professions, the desire to remain in the profession, and awareness of the costs associated with leaving it), professional behavior (9 items, referring to medical students’ behavioral tendencies in medical practice, including professional skills and behavioral characteristics), professional expectation (4 items, referring to the anticipated level of career development and expectations of achieving professional success, including perceptions of the broader context of the medical profession and prospects for individual achievement within this context), and professional values (8 items, referring to medical students’ fundamental views and evaluations of the value of the medical profession, i.e., the manifestation of values within the medical profession). A 5-point Likert scale was used for scoring (1 = strongly disagree, 5 = strongly agree), with four reverse-scored items requiring data transformation. Higher scores on the scale indicate a stronger level of professional identity. This scale has been widely used among Chinese medical student populations and has consistently demonstrated good reliability and validity. When our research team applied it to the resident population, cross-cultural adaptation was performed, confirming that all items maintained content relevance and semantic appropriateness. Related research utilizing this adapted version has been published (19). In the present study, the scale’s Cronbach’s *α* coefficient was 0.879.

### 2.3 Data collection and quality control methods

A questionnaire was created using the Questionnaire Star platform to generate a QR code for online survey distribution. Prior to the survey, coordination was obtained from the directors and staff of the standardized residency training management departments at the participating training bases. The survey link was distributed to first-year residents by their instructors. A unified and detailed informed consent statement was provided at the beginning of the questionnaire, outlining the study’s purpose and procedures, as well as participants’ rights (anonymity, confidentiality, voluntary participation, freedom from harm, and the right to withdraw consent at any time during the study). Participants were required to read and confirm their consent before proceeding. If a participant declined, the questionnaire would automatically close. During data cleaning, responses with completion times of less than 300 s or more than 1,800 s were excluded as outliers.

### 2.4 Statistical analysis

Statistical analysis was performed using SPSS 24.0 software. Categorical variables were described using frequencies and percentages (%), while continuous variables were described using mean ± standard deviation.

Latent profile analysis (LPA) was conducted to identify subgroups of transition shock among first-year residents using R software (version 4.4.1) and relevant packages. The analysis utilized the tidyLPA and tidyverse packages for LPA, supplemented by the mclust package for model fitting and evaluation.

The modeling process began by specifying a 1-class model, incrementally increasing the number of classes. Model fit indices were compared across competing models to select the optimal solution. The following indices were used for model evaluation: Akaike information criterion (AIC) and Bayesian information criterion (BIC): Smaller values indicate better model fit. Entropy: Measures classification accuracy, ranging from 0 to 1. Values closer to 1 indicate higher classification precision; values below 0.6 are generally considered indicative of poor classification quality. Bootstrapped likelihood ratio test (BLRT): Used to compare the fit between nested models (e.g., a *k*-class model vs. a *k* − 1 class model). A significant *p*-value (*p* < 0.05) suggests that the k-class model provides a significantly better fit than the *k* − 1 class model. prob_min and prob_max: Represent the minimum and maximum posterior probabilities of individuals being assigned to their most likely class, respectively. Ideally, prob_min should be greater than 0.7 and prob_max should be close to 1 to ensure reliable classification. n_min and n_max: Represent the proportion of the smallest and largest classes, respectively, among all latent classes, reflecting class balance. Generally, n_min should be greater than 0.1 to ensure sufficient sample size for each class, while n_max should not be excessively large, which might indicate one overly dominant class.

During model selection, theoretical relevance and practical interpretability were considered alongside the statistical indices mentioned above. Furthermore, the proportional sample size of each class was ensured to be reasonable (typically requiring a minimum class proportion ≥10%) to avoid class sparsity or underrepresentation.

Based on the optimal model, chi-square tests and one-way analysis of variance (ANOVA) were employed to compare differences in general characteristics and professional identity across the identified latent classes of first-year residents. Multivariate analysis was performed using multinomial logistic regression. The statistical significance level was set at *α* = 0.05.

## 3 Results

### 3.1 General characteristics

**Figure 1:**
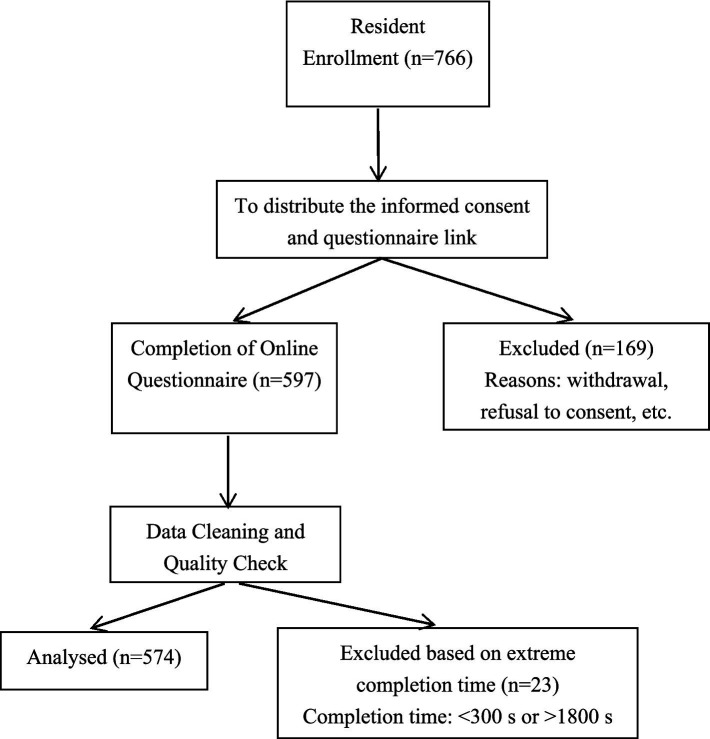
Flow diagram of study participants.

This investigation, conducted from September 2023 to August 2024, employed a cluster sampling method to survey first-year residents from four national-level standardized residency training bases in Hubei Province, China. Out of 766 targeted residents, questionnaires were submitted by 597, yielding a response rate of 77.94%. After excluding responses with extreme completion times, 574 valid questionnaires were retained, resulting in a valid response rate of 96.15%. During the data cleaning process, all variables were checked for missing values, and none were identified. Details are presented in Figure 1.

### 3.2 Transition shock scores among first-year residents

The total score on the Transition Shock Scale for the surveyed participants was 83.43 ± 18.25. The mean scores for the individual dimensions were as follows: physical dimension (3.20 ± 0.82), psychological dimension (2.83 ± 0.80), knowledge and skills dimension (3.59 ± 0.74), and sociocultural and developmental dimension (2.86 ± 0.75).

### 3.3 Results of latent profile analysis on transition shock among first-year residents

Three models were fitted in this study, starting with a single-class model. With increasing class numbers, both AIC and BIC decreased progressively, while the Entropy value increased. The 3-class model demonstrated the smallest AIC and BIC values, an Entropy >0.8, and a statistically significant BLRT. This model exhibited excellent fit indices, more balanced class proportions, and the three identified classes represented distinct combinations of transition shock dimensions, clearly differentiated as high, medium, and low levels. Consequently, the 3-class model was selected as the optimal model for characterizing transition shock among first-year residents. The model fit indices are presented in Table 1.

**Table 1:** Fit indices for latent profile analysis models (1–3 classes).

Classes	AIC	BIC	Entropy	Prob_min	Prob_max	n_min	n_max	BLRT_*p*
1	45048.80	45283.84	1.00	1.00	1.00	1.00	1.00	
2	41191.76	41666.20	0.92	0.98	0.98	0.47	0.53	0.01
3	39407.70	40121.53	0.92	0.96	0.97	0.24	0.49	0.01

AIC, Akaike information criterion; BIC, Bayesian information criterion; BLRT_*p*, bootstrap likelihood ratio test *p*-value; entropy indicates classification accuracy; prob_min, the minimum posterior classification probability among all individuals, representing the least certain classification in the sample; prob_max, the maximum posterior classification probability among all individuals, representing the most certain classification in the sample; n_min and n_max indicate the proportion of individuals in the smallest and largest classes, respectively. The 3-class solution was selected as the optimal model based on lower AIC/BIC values, acceptable entropy, and significant BLRT.

**Figure 2:**
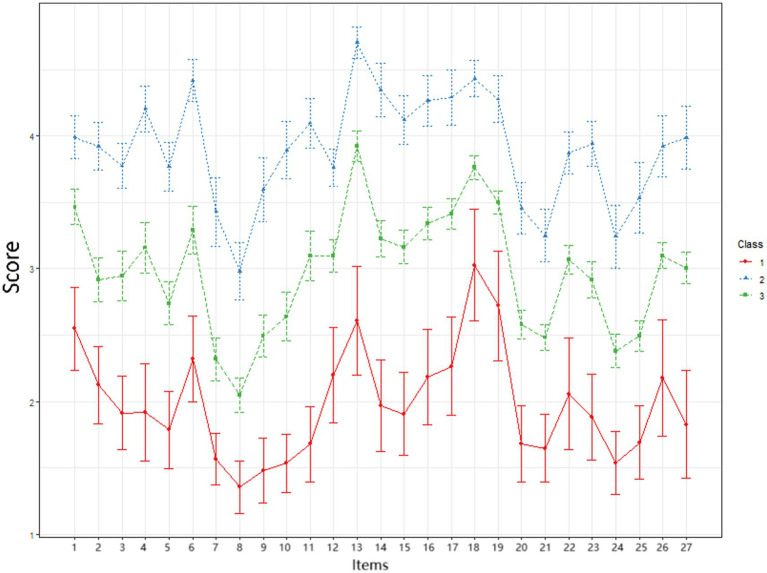
Latent profiles of transition shock among first-year residents (*n* = 574). The *x*-axis represents the 27 measured variables (items a to aa). The *y*-axis shows the standardized mean scores (*z*-scores) for each profile. The three colored lines represent the distinct response patterns of the three latent classes. The vertical error bars indicate the standard errors of the mean estimates for each item within each profile.

The three classes identified in this study were named based on the participants’ scores on the individual items of the Transition Shock Scale. Group 1 had lower scores across all items compared to the other two groups, with notably low scores in the “psychological” and “sociocultural and developmental” dimensions; it was therefore named the “low psychological-sociocultural shock” group, comprising 77 individuals (13.41%). Group 2 had the highest scores across all items, with the highest-scoring items primarily concentrated in the “physical” and “knowledge and skills” dimensions; it was consequently named the “high physical-knowledge/skill shock” group, comprising 155 individuals (27.01%). Group 3 had scores across all dimensions that fell between those of Group 1 and Group 2; it was named the “moderate transition shock” group, comprising 342 individuals (59.58%). The characteristic profiles of the three latent transition shock classes are shown in Figure 2.

### 3.4 Univariate analysis of latent transition shock profiles among first-year residents

General characteristics and professional identity scores were compared across the three latent classes of residents; the results are presented in Table 2. Resident gender, age, ethnicity, and marital status showed no significant association with latent class membership (*p* > 0.05). Statistically significant differences were observed among the latent classes for training status and the scores on the professional identity dimensions: professional cognition, professional emotion, professional commitment, professional behavior, professional expectation, and professional values (*p* < 0.05).

**Table 2:** Univariate analysis of general characteristics and professional identity scores across latent classes of transition shock in first-year residents.

Variables	Subgroups	*n*	Low psychological-sociocultural shock group (*n* = 77)	Moderate transition shock group (*n* = 342)	High physical-knowledge/skill shock group (*n* = 155)	*F* value	*p*-value
Gender						3.823	0.148
	Male	252	41 (16.2%)	141 (56.0%)	70 (27.8%)		
	Female	322	36 (11.2%)	201 (62.4%)	85 (26.4%)		
Age						7.242	0.124
	<25	391	48 (12.3%)	232 (59.3%)	111 (28.4%)		
	25–30	167	24 (14.4%)	100 (59.9%)	43 (25.7%)		
	>30	16	5 (31.3%)	1 (6.2%)	10 (62.5%)		
Ethnicity						0.594	0.743
	Han	472	61 (12.9%)	282 (59.8%)	129 (27.3%)		
	Minority	102	16 (15.7%)	60 (58.8%)	26 (25.5%)		
Training status						27.052	<0.001
	In-house resident trainees	19	3 (15.8%)	14 (73.7%)	2 (10.5%)		
	Master of Medicine (MM) degree candidates	300	25 (8.3%)	174 (58.0%)	101 (33.7%)		
	Externally commissioned resident trainees	57	7 (12.3%)	37 (64.9%)	13 (22.8%)		
	Non-affiliated resident trainees	198	42 (21.2%)	117 (59.1%)	39 (19.7%)		
Marital status						2.890	0.236
	Unmarried	539	69 (12.8%)	323 (59.9%)	147 (27.3%)		
	Married	35	8 (22.9%)	19 (54.2%)	8 (22.9%)		
Professional identity							
	Professional cognition	20.26 ± 3.71	23.32 ± 4.08	20.37 ± 3.13	18.49 ± 3.65	51.967	<0.001
	Professional emotion	24.30 ± 4.75	27.86 ± 5.59	24.32 ± 3.98	22.51 ± 4.88	36.753	<0.001
	Professional commitment	11.36 ± 2.10	12.70 ± 2.17	11.47 ± 1.92	10.45 ± 2.05	34.393	<0.001
	Professional behavior	31.09 ± 4.91	34.77 ± 6.46	31.03 ± 4.13	29.42 ± 4.67	34.067	<0.001
	Professional expectation	14.27 ± 2.65	16.53 ± 3.12	14.31 ± 2.28	13.06 ± 2.41	51.879	<0.001
	Professional values	28.38 ± 5.00	31.75 ± 5.98	28.27 ± 4.43	26.95 ± 4.92	26.029	<0.001

### 3.5 Regression analysis of latent transition shock classes among first-year residents

Using the three latent classes as the dependent variable, variables showing statistical significance in the univariate analysis were included in the multinomial logistic regression model. The results demonstrated that, when comparing Group 2 (“high physical-knowledge/skill shock” group) versus Group 1 (“low psychological-sociocultural shock” group) using Group 1 as the reference, Master of Medicine (MM) degree candidates were more likely to be classified into Group 2 compared to non-affiliated resident trainees. Higher scores in professional cognition, professional commitment, and professional expectation were associated with a higher likelihood of belonging to Group 1. Conversely, higher professional values scores were associated with an increased likelihood of belonging to Group 2.

When comparing Group 1 versus Group 3 (“moderate transition shock” group) using Group 3 as the reference, higher professional cognition scores were associated with a greater likelihood of belonging to Group 1, while MM degree candidates showed a higher probability of being classified into Group 3.

When comparing Group 2 versus Group 3 using Group 3 as the reference, higher scores in professional cognition, professional commitment, and professional expectation were associated with a greater likelihood of belonging to Group 3, whereas higher professional values scores increased the probability of classification into Group 2. The complete results of the multinomial logistic regression are presented in Table 3.

**Table 3:** Results of multinomial logistic regression analyzing factors associated with latent transition shock classes in first-year residents.

Group comparison	Variables	*β*	SE	Wald	*p*-value	OR value (95% CI)
Group 2 vs. Group 1 ①	Intercept	8.855	1.980	19.993	<0.001	—
	Professional cognition	−0.323	0.064	25.580	<0.001	0.724 (0.639–0.820)
	Professional commitment	−0.235	0.111	4.439	0.035	0.791 (0.636–0.984)
	Professional expectation	−0.321	0.109	8.687	0.003	0.725 (0.586–0.898)
	Professional values	0.130	0.059	4.841	0.028	1.139 (1.014–1.279)
	Master of medicine candidates	1.246	0.363	11.775	0.001	3.477 (1.707–7.086)
Group 1 vs. Group 3②	Intercept	−7.780	1.448	28.857	<0.001	—
	Master of medicine candidates	−0.806	0.309	6.813	0.009	0.447 (0.244–0.818)
	Professional cognition	0.155	0.055	8.008	0.005	1.168 (1.049–1.301)
Group 2 vs. Group 3②	Intercept	1.075	1.503	0.512	0.474	
	Professional cognition	−0.168	0.041	17.072	<0.001	0.846 (0.781–0.916)
	Professional commitment	−0.210	0.073	8.229	0.004	0.811 (0.702–0.936)
	Professional expectation	−0.155	0.071	4.798	0.028	0.856 (0.745–0.984)
	Professional values	0.079	0.035	5.231	0.022	1.082 (1.011–1.158)

Group 1: “Low psychological-sociocultural shock” group.

Group 2: “High physical-knowledge and skills shock” group.

Group 3: “Moderate transition shock” group.

① Indicates comparison with Group 1. ② Indicates comparison with Group 3.

## 4 Discussion

### 4.1 Multidimensional characteristics and population heterogeneity of resident transition shock

This study found that the total transition shock score among first-year residents was 83.43 ± 18.25, indicating a moderate level of transition shock. The highest score was observed in the knowledge and skills dimension (3.59 ± 0.74), while the sociocultural and developmental and psychological dimensions scored relatively lower (2.86 ± 0.75 and 2.83 ± 0.80, respectively). These results suggest that the most significant challenge during the transition period stems from stress related to unskilled operational procedures, sudden changes in patient conditions, and feelings of helplessness in clinical work. This may be attributed to the primary pressure faced by residents during the early stages of standardized training—the urgent need to rapidly develop clinical competency. In contrast, sociocultural adaptation, as part of the hidden curriculum, often exhibits a delayed impact. The discrepancy between the psychological and physical dimension scores (3.20 ± 0.82) implies that the immediate perception of physiological strain may take precedence over the activation of emotional regulation mechanisms.

Based on the latent profile analysis, this study identified three distinct classes of transition shock among first-year residents. The model demonstrated good fit, indicating significant heterogeneity in transition shock experiences among newly enrolled residents. Approximately 59.58% of residents belonged to the “moderate transition shock” group. The “low psychological-sociocultural shock” group demonstrated low scores across all dimensions. This population may possess richer personal resources—potentially through higher psychological capital, pre-adaptation mechanisms (such as clinical internship experience during undergraduate education), or robust social support—enabling them to navigate or complete the “professional socialization” process more smoothly and effectively buffer transition stress. Although this group comprised only 13.41% of residents, further exploration of their protective factors holds significant implications for designing future interventions. Conversely, the “high physical-knowledge/skill shock” group (27.01%) experienced the highest levels of impact in physical strain and knowledge/skill dimensions, precisely aligning with the health impairment pathway of the JD-R model: they face excessive job demands—managing high-intensity clinical rotations while rapidly mastering diagnostic and treatment protocols—leading to a cumulative stress effect (39–41). However, their personal resources (e.g., clinical competency, coping experience) or social support may be relatively insufficient, resulting in intense shock experiences.

### 4.2 Differential moderating effects of professional identity components

Multivariate analysis revealed significant differentiation in how various dimensions of professional identity predicted transition shock class membership: higher scores in professional cognition, professional commitment, and professional expectation were associated with an increased likelihood of belonging to the “low psychological-sociocultural shock” group. This suggests that residents with a clearer understanding of their profession, stronger professional commitment, and more realistic career expectations were more likely to experience milder transition shock, potentially due to possessing stronger motivation to overcome difficulties and cope with transitional challenges.

The study also observed a noteworthy phenomenon warranting further investigation: paradoxically, higher scores in professional values were associated with an increased risk of belonging to the “high physical-knowledge/skill shock” group. To control for potential confounding factors, we calculated the variance inflation factor (VIF) for each dimension of professional identity. All VIF values were below 4, indicating no evidence of multicollinearity and supporting the association between higher professional values and membership in the “high physical-knowledge/skill shock” group. Integrating characteristics of clinical training, we posit that this pattern may occur because professional values, being more closely tied to internal beliefs, influence intrinsic motivation and satisfaction. This data pattern may be attributed to the following reasons: (1) Residents with strong professional values hold higher professional ideals. This internalized value system itself constitutes a form of “demand.” When reality falls short, they are prone to an “idealism-reality gap”—where expectations for excellence and service conflict with the practical constraints faced during their first year, potentially creating moral distress and self-perceived overload, thereby triggering deeper internal conflict and stress. Within the job demands-resources (JD-R) model framework, internalized values elevate internal demands (self-expectations, sense of responsibility), which, when compounded by external high job demands and insufficient resources (e.g., guidance, protected time, structured feedback), can intensify shock and pressure. Future studies should examine this pathway by incorporating measures of moral distress and evaluate whether cognitive restructuring and reflective supervision can mitigate this effect; (2) Strong professional values may be accompanied by a greater sense of responsibility, potentially amplifying internal pressure when facing challenges and thereby intensifying transition shock; (3) Trainees with strong professional values might find it more difficult to accept necessary compromises in real-world settings, facing greater difficulties in adapting to the new environment. These interpretations are derived from integrative inferences based on existing theories, clinical teaching experience, and the findings of this study. Their precise underlying mechanisms and psychological pathways require further empirical verification in future research.

### 4.3 Greater transition shock among master of medicine degree candidates

Master of Medicine (MM) degree candidates experienced the greatest transition shock during their role transition from medical student to resident physician within standardized training bases. Using the low psychological-sociocultural shock group as the reference, the phenomenon that resident physicians with Master of Medicine degree candidate status are more likely to be classified into the “high physical-knowledge/skill shock” group compared to socially recruited resident trainees highlights the stressor of “dual-role conflict.” These individuals maintain their student status, continuing to fulfill academic requirements and research obligations for their master’s degree, while simultaneously commencing clinical rotations in training bases. They must integrate theoretical knowledge with clinical practice and assume the responsibilities of a resident physician. This dual identity as both graduate student and resident requires constant balancing of academic research and clinical practice. Such role overload implies exposure to greater pressures and challenges (42), which may explain their higher levels of transition shock.

### 4.4 Targeted pathways for educational intervention

For first-year residents, regardless of their specific trainee classification, adapting to the resident physician role requires time to familiarize themselves with the standardized residency training system and rotation requirements. As they accumulate experience in specialized disease assessment and treatment plan selection, the inherent responsibilities of the resident role can simultaneously induce psychological and professional knowledge/skill shocks.

Based on the three-class model identified in this study, administrators and faculty of standardized residency training should understand and respect individual differences among residents from various backgrounds. They need to recognize that these individuals demonstrate distinct characteristics in psychological, physical, knowledge/skill, and cultural-developmental adaptation during their professional role transition and adjustment to the new environment. Differentiated management and precise intervention strategies should be implemented according to their specific needs: For the “high physical-knowledge/skill shock” group, the core conflict lies between high-intensity clinical tasks and deficiencies in knowledge/skills. Interventions should focus on rapidly enhancing clinical competence and rationally distributing workloads. A tiered competency training model can be implemented: during initial training, emphasize high-frequency clinical procedures and emergency response protocols to strengthen fundamental clinical skills. For example, one physician licensure-related procedural skill is trained every 2 weeks. During the first-year rotations, residents complete all training and assessments covering the required clinical skills for the Chinese Medical Practitioner Qualification examination. Standardized assessments utilize technical skills checklists and the direct observation of procedural skills (DOPS) framework. Trainees should operate under direct supervisor guidance until passing departmental assessments before gradually increasing workload. Provide timely feedback and targeted support for specific skill difficulties. This structured skill training combined with progressive workload adjustment helps alleviate somatic manifestations of stress. For the high-risk group motivated by strong professional values, particular attention should be paid to the disparity between their professional ideals and complex clinical reality. Facilitate cognitive restructuring and emotional regulation through monthly sessions led by senior physicians, guiding residents to share experiences of frustration and achievement. These sessions should help residents learn to derive motivation and meaning from challenges. By sharing their own experiences with clinical hardships and realistic constraints, senior physicians can help transform idealism into pragmatic professionalism. Furthermore, the special needs of Master of Medicine degree candidates indicate the necessity to establish a dual-track “academic-clinical” support system. Enhance the collaborative mechanism between research supervisors and clinical mentors through regular communication (such as organizing monthly dual-mentor meetings) and jointly coordinated scheduling of clinical and academic activities, thereby alleviating role overload among these residents. Administrators and faculty must fully understand the distinct characteristics of residents from different backgrounds. While ensuring standardized training, they should implement differentiated management strategies to assist residents in successfully navigating the transition period, thereby facilitating the enhancement of their social identity and professional status.

## 5 Study limitations and future directions

This study has several limitations. First, the cross-sectional design can reveal associations between variables but cannot establish causality or capture the dynamic evolution of transition shock. Therefore, the discussions regarding the dimensions of professional identity and the degree of transition shock in this paper refer solely to correlational relationships. Future studies could employ longitudinal LPA to investigate transition pathways among shock categories. Second, although multiple national-level training bases were selected to enhance sample representativeness, all participants were from Hubei Province, China, which may affect the generalizability of the classification model. Subsequent research should conduct multi-center validation across different regions and incorporate qualitative interviews to deeply analyze coping strategies among various subgroups. Furthermore, cluster sampling may introduce clustering effects, which were not adjusted for in the statistical analysis of this study. Third, although the Professional Identity and Transition Shock scales used in this study underwent cultural adaptation, their psychometric properties warrant further verification. Future research could develop specialized assessment tools specifically tailored for the resident population. Finally, the data were derived from self-reports, which may be subject to inaccuracies, subjectivity, recall bias, potential common method bias, and social desirability effects. The online survey method might also introduce self-selection bias.

An additional limitation is that the specific clinical rotations of the participants were not considered. As this study focused on first-year residents in the early stages of standardized training, who were still undergoing rotational assignments, the study design did not collect or analyze departmental information. Practical contextual factors (such as differences between departments, teaching styles of supervisors, and work environments) may act as potential confounders. Future research should incorporate participants’ fixed departmental assignments as an important factor in the study design to assess its potential impact.

## 6 Conclusion

This study identified three distinct transition shock profiles among first-year residents in China using latent profile analysis: “low psychological-sociocultural shock,” “high physical-knowledge/skill shock,” and “moderate transition shock.” Different dimensions of professional identity demonstrated specific associations with these transition shock profiles. Higher levels of professional cognition, commitment, and expectations were associated with milder transition shock, whereas stronger professional values potentially served as a risk factor for “high physical-knowledge/skill shock,” possibly mediated by an idealism-reality gap. Master of Medicine degree candidates faced increased transition shock risk due to role overload.

These findings carry clear practical implications for residency training management: implementing a tiered competency-building model for the “high physical-knowledge/skill shock” group; providing cognitive restructuring and emotional support for the high-risk group driven by strong professional values; and establishing a dual-track support system with collaborative supervision between academic and clinical mentors for Master of Medicine degree candidates. Future interventions should be specifically tailored to these distinct profiles to improve training outcomes and psychological well-being among residents.

## Data Availability

The raw data supporting the conclusions of this article will be made available by the authors, without undue reservation.
