# Correction: A study on transition shock among first-year residents in China: survey data based on latent profile analysis

**DOI:** 10.3389/fmed.2025.1768609

**Published:** 2026-01-12

**Authors:** Di Xue, Huaijie Yang, Jian Zheng, Aijun Zhu

**Affiliations:** 1 The First College of Clinical Medical Science, China Three Gorges University, Yichang, China; 2 College of Medicine and Health Sciences, China Three Gorges University, Yichang, China; 3 Yichang Central People's Hospital, Yichang, China

**Keywords:** residents, standardized residency training, transition shock, latent profile analysis, professional identity

The order of affiliations in the affiliation list of the published paper was erroneously given as: ^1^College of Medicine and Health Sciences, China Three Gorges University, Yichang, China, ^2^Yichang Central People's Hospital, Yichang, China, ^3^The First College of Clinical Medical Science, China Three Gorges University, Yichang, China.

The correct author list reads:

^1^The First College of Clinical Medical Science, China Three Gorges University, Yichang, China, ^2^College of Medicine and Health Sciences, China Three Gorges University, Yichang, China, ^3^Yichang Central People's Hospital, Yichang, China.

Affiliation The First College of Clinical Medical Science, China Three Gorges University, Yichang, China was omitted for author Di Xue. This affiliation has now been added for author Di Xue.

Affiliation The First College of Clinical Medical Science, China Three Gorges University, Yichang, China was omitted for author Aijun Zhu. This affiliation has now been added for author Aijun Zhu.

The original version of this article has been updated.

